# The Relationship Between Self-Concept Clarity and Meaning in Life Among Adolescents: Based on Variable-Centered Perspective and Person-Centered Perspective

**DOI:** 10.3390/bs15070948

**Published:** 2025-07-14

**Authors:** Yang Yang, Hongyu Wang, Shaoying Gong, Yin Qiu

**Affiliations:** 1 Mental Health Education Center, Huanghuai University, Zhumadian 463000, China; 2 Key Laboratory of Adolescent Cyberpsychology and Behavior (CCNU), Ministry of Education, Wuhan 430079, China; 3 Key Laboratory of Human Development and Mental Health of Hubei Province, School of Psychology, Central China Normal University, Wuhan 430079, China; 4 Mental Health Education Center, Wuhan College, Wuhan 430212, China

**Keywords:** self-concept clarity, meaning in life, latent profile analysis (LPA)

## Abstract

A clear understanding of one’s identity is essential for adolescents to lead a meaningful and purposeful life. We investigated the relationship between self-concept clarity and meaning in life among 2288 adolescents through a cross-sectional survey, using both variable-centered and person-centered approaches. The results revealed a significant positive correlation between self-concept clarity and meaning in life among Chinese adolescents, as well as its dimensions. Three distinct profiles of self-concept clarity were identified among adolescents: high, medium, and low. The profile with high self-concept clarity had the highest meaning in life, followed by the moderate self-concept clarity profile, while the low self-concept clarity group had the lowest meaning in life. The findings underscore the enduring impact of self-concept clarity on adolescents’ meaning in life, affirming its central function within the self-concept structure in shaping meaning in life.

## 1. Introduction

Meaning in life is an important predictor of physical and mental health (Czekierda et al., 2017; Kim et al., 2019; Roepke et al., 2014; Steger, 2017; Zhao et al., 2017). Viktor Frankl, the founder of logotherapy, asserts that numerous psychological distress and mental health problems frequently stem from a fundamental absence of meaning in life (Steger et al., 2008). The absence of meaning in life might result in feelings of emptiness, boredom, depression (H. Park & Jeong, 2016), post-traumatic stress disorder (Fischer et al., 2020), and even behaviors such as self-harm and suicide (Costanza et al., 2019; Ilagan, 2023).

More researchers are recognizing the positive influence of meaning in life (Steger et al., 2008). Individuals with a high level of meaning in life tend to experience greater well-being (Krok, 2018), stronger psychological resilience (Lin, 2021), and better academic performance (Makola, 2014; Mason, 2017). Meanwhile, meaning in life also serves a protective function, effectively alleviating depression and anxiety in adolescents (Korkmaz & Güloğlu, 2021; Tsibidaki, 2021), and acting as an important buffer against everyday stress (J. Park & Baumeister, 2017). Following traumatic events, individuals with high meaning in life typically report greater use of effective coping strategies and faster recovery from trauma (Jim et al., 2006). Therefore, guiding adolescents to develop a proper understanding of life’s value and encouraging them to pursue its meaning carries significant theoretical and practical implications.

For adolescents, having a clear understanding of who they are is essential for leading a meaningful and purposeful life (Thomas et al., 2022). Self-concept clarity plays a crucial role in regulating cognition, emotion, and behavior within self-involving contexts. Therefore, its influence on adolescents’ meaning in life should not be overlooked, as it serves as a powerful source of meaning in life (Boucher et al., 2016).

Most existing research on the association between self-concept clarity and meaning in life has been grounded in the two-dimensional conceptualization of meaning in life. Measurement tools have mostly focused on the “presence of meaning” and “search for meaning” (Steger et al., 2006). Thus, although prior research has demonstrated a positive link between self-concept clarity and meaning in life, the relationship has been assessed largely through individuals’ intuitive perceptions of meaning (Hicks & King, 2009), potentially overlooking the deeper structure of meaning in life. In recent years, researchers have found that the three-dimensional concept of meaning in life goes beyond merely focusing on the surface of “meaning” and more deeply reflects the intrinsic characteristics of meaning in life (Costin & Vignoles, 2020; George & Park, 2017; King & Hicks, 2021; Martela & Steger, 2016). With the adoption of the three-dimensional framework, meaning in life is now understood to comprise three key dimensions: coherence, purpose, and significance (George & Park, 2017; Martela & Steger, 2016). Coherence refers to individuals’ perceptions of the consistency of their lives and the extent to which they feel understood. Purpose refers to the extent to which individuals perceive their lives as being guided and motivated by meaningful goals. Significance refers to the extent to which individuals feel that their existence holds value. This expanded perspective provides an opportunity to explore how self-concept clarity might relate differently to each dimension, thus enriching our understanding of the effect through which self-concept clarity contributes to meaning in life.

Self-concept clarity might influence individuals’ perception of the coherence of meaning in their life experiences. Self-Verification Theory suggests that individuals are motivated to seek, accept, and retain information that aligns with their existing self-concept, while discounting or resisting information that is inconsistent with it (Swann, 1997). Therefore, self-concept clarity is essential for how individuals experience and perceive the world. Self-concept clarity facilitates the establishment and maintenance of a stable identity (Gregg & Sedikides, 2018; Pilarska, 2016; Schwartz et al., 2011), which in turn supports individuals in constructing meaningful life narratives through self-storytelling, integrating life events into a coherent whole. Self-concept clarity helps individuals expand their self in relational domains, providing additional sources of relational support for coherent life experiences (Emery et al., 2015). This, in turn, promotes healthier and more stable social support, thereby introducing an interpersonal dimension to the coherence of life. Additionally, individuals with high self-concept clarity are more adaptable and can flexibly respond to changes in life (Miyagawa, 2022), allowing them to adjust their behaviors and goals to fit new situations, thereby maintaining coherence. In contrast, uncertainty about one’s identity might have the opposite effect, ultimately undermining the perception of coherence (George & Park, 2016). For example, low self-concept clarity directly undermines an individual’s self-continuity and can also impair self-continuity by reducing the role of self-reflection and self-narrative in autobiographical memory (Jiang et al., 2023), ultimately leading to a decline in meaning in life (Chu & Lowery, 2024). Therefore, self-concept clarity might influence the perceived coherence of life.

Self-concept clarity might influence individuals’ perception of pursuing valuable goals and establishing clear directions in life. According to the model proposed by Light (2017) on the role of self-concept clarity in goal pursuit, self-concept clarity might play a role in the pre-decision, post-decision, pre-action, and action stages of goal pursuit. In the pre-decision stage, high self-concept clarity helps individuals set clear and achievable goals. Low self-concept clarity might lead individuals to struggle with determining what they truly want, thereby reducing goal clarity. Previous research has shown that individuals with high self-concept clarity perform better in social decision-making, while those with lower self-concept clarity tend to show indecisiveness, leading to poorer social decisions (Uğurlar & Wulff, 2022). In the post-decision and pre-action stages, individuals with high self-concept clarity have a clearer understanding of their strengths and weaknesses. They consider their abilities and limitations and seek accurate information about themselves to support goal achievement. Adolescents with high self-concept clarity are more optimistic, have a longer-term perspective on the future (Morawiak et al., 2018; Steger et al., 2009), are more willing to invest effort in learning, exhibit greater persistence (Gupta & Bakker, 2020), and are more likely to persist in pursuing their goals while resisting temptations (Jiang et al., 2023). In the action stage, individuals primarily focus on taking concrete steps toward their goals. Individuals with high self-concept clarity are more likely to adopt proactive coping strategies in the process of goal achievement (Smith et al., 1996) and exhibit greater persistence (Fite et al., 2017; Wong & Vallacher, 2018). At the same time, individuals with high self-concept clarity are better able to focus their attention and determination while pursuing goals, making them less susceptible to external distractions (Jiang et al., 2023), which increases their goal achievement and purpose in life. In contrast, individuals with low self-concept clarity might adopt more passive coping strategies (Smith et al., 1996), hindering goal attainment and leading to a decrease in their sense of purpose in life. Therefore, self-concept clarity might influence the development and maintenance of an individual’s sense of purpose.

Self-concept clarity might influence individuals’ perception of their self-worth and significance. Firstly, self-concept clarity is associated with a more confident, internally consistent, and accessible self-view, which makes individuals less sensitive to external feedback, particularly negative feedback (Guerrettaz et al., 2014). Lower sensitivity to negative feedback could help individuals cultivate and maintain stronger significance. Research has also shown that individuals with higher self-concept clarity have a stronger association with self-worth (Ferris et al., 2009; Koriat, 2012; B. Zhang & Lin, 2023). Secondly, self-concept clarity could influence individuals’ perceived significance by affecting their true self. Individuals with high self-concept clarity are more likely to experience their true self (Boucher, 2011, 2021; Kraus et al., 2011). The true self reflects alignment between an individual’s behavior and their unique sources of significance, thereby increasing the perceived importance (Lutz et al., 2023). Moreover, self-esteem, a psychological construct closely related to self-concept clarity (Weber et al., 2023), is strongly associated with perceived significance. High self-esteem helps individuals develop positive self-cognitions, viewing themselves as valuable and meaningful. In conclusion, self-concept clarity might influence individuals’ perceived importance.

Moreover, in previous studies examining the relationship between self-concept clarity and meaning in life, most have assessed self-concept clarity based on the total or average scores. However, this method often overlooks the heterogeneity within groups (Xiang, 2022), which might lead to inaccurate categorization. The person-context interaction theory proposes that complex environments may lead to diversity as a fundamental feature of human development. Individuals with different characteristics tend to cluster together based on similar combinations of psychological and behavioral traits, thus forming relatively stable “types”(Magnusson & Stattin, 1998). In line with the theory, individual differences in self-concept clarity may also demonstrate distinct patterns, suggesting the presence of diverse subgroups. Therefore, to explore heterogeneity in participants’ self-concept clarity, latent profile analysis was used to classify individuals into distinct groups based on their response patterns (Q. Li et al., 2022; Xiang, 2022). Studies have demonstrated that adolescents’ self-concept clarity existed in three profiles: high, medium, and low.

Previous research has shown that indicated that self-concept clarity varies by age and gender; but the results are inconsistent (Crocetti et al., 2016; Lodi-Smith et al., 2017; Xiang et al., 2023). The findings on age and gender differences in self-concept clarity vary, possibly due to cultural differences, limited sample representativeness, or small sample sizes. In addition, only one study has indicated that age and gender influence the profiles of self-concept clarity (Xiang et al., 2021). Therefore, the study aims to provide new evidence supporting the impact of age and gender on the profiles of self-concept clarity.

Meanwhile, individuals with different profiles of self-concept clarity experience differential levels of subjective well-being subjective well-being (Xiang, 2022). However, a compelling and underexplored question is whether individuals characterized by different profiles of self-concept clarity experience distinct levels of meaning in life.

Therefore, within the framework of the three-dimensional conceptualization of meaning in life, the study adopts both variable-centered and person-centered approaches to examine the relationship between self-concept clarity and adolescents’ meaning in life. The former explores the overall association between the two constructs, while the latter investigates how different profiles of self-concept clarity relate to adolescents’ meaning in life.

The specific hypotheses are as follows:

**H_1_.**  *Self-concept clarity is positively associated with adolescents’ meaning in life and its various dimensions.*

**H_2_.**  *Age and gender are significantly associated with the profiles of self-concept clarity.*

**H_3_.**  *Self-concept clarity exists in different profiles, and adolescents’ meaning in life varies across these profiles.*

## 2. Materials and Methods

### 2.1. Participants

A cluster sampling strategy was adopted, and data were gathered from three middle schools and one university across three different cities in Henan Province, comprising a total of 2288 adolescents. Among them, 540 were junior middle school students, with the average age of 13.73 years (*SD* = 0.77). Of the junior middle school students, 282 were male, 258 were female, and 512 were non-only children (2 participants did not respond). In terms of residential areas, 130 participants were from urban areas, 186 were from towns, and 221 were from rural areas, with 3 participants not providing this information. Among them, 634 were senior high school students, with the average age of 16.61 years (*SD* = 0.71). Among the senior high school students, 234 were male, 400 were female, and 609 were non-only children (4 participants did not provide a response). Regarding residential areas, 77 participants were from urban areas, 130 from towns, and 422 from rural areas, with 5 participants not reporting this information. The university sample consisted of 1114 students, with a mean age of 18.47 years (*SD* = 0.89). Of these, 466 were male, 648 were female, and 983 were non-only children. In terms of residential areas, 248 participants were from urban areas, 234 from towns, and 632 from rural areas.

### 2.2. Measures

Self-Concept Clarity: the scale revised by Niu et al. (2016), based on the original measure developed by Campbell et al. (1996), was used. It consists of 12 items (sample item: if I were to describe my personality, perhaps my description would be different every day) with all items being reverse-scored except for items 6 and 11. The scale used a 5-point Likert scale (1 = strongly disagree, 5 = strongly agree), with higher scores indicating higher levels of self-concept clarity. The scale has demonstrated good reliability and validity in Chinese samples (Niu et al., 2016; D. Zhang, 2018). The structural validity in the study was satisfactory, χ^2^_(49)_ = 317.27, RMSEA = 0.05, CFI = 0.95, TLI = 0.93, SRMR = 0.03, Cronbach’s α was 0.82.

Meaning in life: we used the Multidimensional Meaning in Life Scale (George & Park, 2017), translated by Zhou et al. (2018). The scale consists of 15 items, including three dimensions: coherence (sample item: I can make sense of the things that happen in my life), purpose (sample item: I have aims in my life that are worth striving for), and significance (sample item: I am certain that my life is of importance), with 5 items for each dimension. It uses a 7-point Likert scale (1 = strongly disagree; 7 = strongly agree), with higher scores indicating higher meaning in life. The results showed that the original scale had good structural validity, χ^2^_(73)_ = 618.46, RMSEA = 0.06, CFI = 0.96, TLI = 0.94, SRMR = 0.03. The Cronbach’s α coefficients for the scale and its dimensions were 0.94, 0.84, 0.90, and 0.85.

### 2.3. Research Procedure

In compliance with the principle of informed consent, and after obtaining approval from both the school and the students, a cluster sampling method was employed for the survey. Given the practical conditions of junior and senior high school students, paper-based questionnaires were used. Class teachers were responsible for administering the survey and distributing and collecting the questionnaires during class. To optimize the process, college students completed the survey via Wenjuanxing, with the class counselors and psychology representatives jointly overseeing the administration, distributing survey links. Prior to data collection, the researcher provided a standardized reading of the informed consent form to all participants, thoroughly explaining the purpose of the study, clarifying its scientific basis, and assuring that all responses would remain strictly confidential and be used exclusively for academic research. To ensure the authenticity of responses, participants were informed that there were no correct or incorrect answers and were encouraged to answer honestly based on their personal experiences.

### 2.4. Data Analysis

Descriptive statistics, correlation analysis, and group comparison tests were conducted using SPSS 25.0, while latent profile analysis was performed using Mplus 8.0. The model fit indices were primarily evaluated based on three categories. The first category includes information criteria, such as the Akaike Information Criterion (AIC), Bayesian Information Criterion (BIC), and the sample size adjusted Bayesian Information Criterion (aBIC), with smaller values indicating better fit. The second category includes Entropy, which represents the accuracy of classification. The range of values is from 0 to 1, with values closer to 1 indicating greater accuracy. A value below 0.6 suggests that more than 20% of individuals are misclassified, while a value above 0.8 indicates that the classification accuracy exceeds 90%. Specifically, a value greater than 0.8 indicates high classification accuracy (Jung & Wickrama, 2008). The third category includes likelihood ratio indices, such as the Vuong-Lo-Mendell-Rubin adjusted likelihood ratio test (VLMR-LRT), which indicates whether the model with K categories fits better than the model with K-1 categories. In addition to relying on the aforementioned model fit indices to determine the optimal number of categories, prior research and theoretical considerations should also be taken into account to finalize the most appropriate classification solution (Nylund et al., 2007).

## 3. Results

### 3.1. The Development of Adolescents’ Self-Concept Clarity and Meaning in Life

The development of self-concept clarity and meaning in life in adolescents is shown in Table 1. First, self-concept clarity (*F*(2, 2282) = 51.51, *p* < 0.001, $\eta_{p}^{2}$ = 0.04), meaning in life (*F*(2, 2282) = 53.78, *p* < 0.001, $\eta_{p}^{2}$ = 0.05), coherence (*F*(2, 2282) = 55.66, *p* < 0.001, $\eta_{p}^{2}$ = 0.05), purpose (*F*(2, 2282) = 57.49, *p* < 0.001, $\eta_{p}^{2}$ = 0.05), and significance (*F*(2, 2282) = 31.73, *p* < 0.001, $\eta_{p}^{2}$ = 0.03) were different among different age groups. Specifically, junior middle school students and senior high school students have lower self-concept clarity compared to college students, but there was no significant difference between junior middle and senior high school students. In terms of overall meaning in life, as well as the dimensions of coherence and purpose, senior high school students scored lower than junior middle school students, who in turn scored lower than college students. Regarding the dimension of significance, college students reported higher levels than both junior middle school and senior high school students, among whom no significant difference was found. Furthermore, both self-concept clarity and meaning in life showed gender differences. Specifically, male adolescents had significantly higher self-concept clarity than female adolescents (*t* = 6.02, *p* < 0.001, d = 0.24). Male adolescents also scored higher than female adolescents on meaning in life (*t* = 2.77, *p* < 0.001, d = 0.12), coherence (*t* = 4.55, *p* < 0.001, d = 0.19), and purpose (*t*= 3.02, *p* < 0.001, d = 0.12); however, no gender differences were found regarding significance (*t* = 0.22, *p* > 0.05). Finally, there were no significant differences in self-concept clarity or meaning in life between only children and non-only children (*p* > 0.05). Additionally, the impact of geographic origin on adolescents’ self-concept clarity and meaning in life was also not significant (*p* > 0.05).

### 3.2. Correlation Analysis of Self-Concept Clarity and Meaning in Life in Adolescents

The results of the correlation analysis were presented in Table 2. The higher the self-concept clarity, the higher the meaning in life experienced by adolescents. Specifically, self-concept clarity was moderately positively correlated with meaning in life and with each of the dimensions (*r* = 0.35–0.49).

### 3.3. Latent Profile Analysis and Characteristics of Self-Concept Clarity in Adolescents

To identify latent subtypes of self-concept clarity, latent profile analysis was conducted on the items of the Self-Concept Clarity Scale. From a person-centered perspective, the study further examined differences in adolescents’ meaning in life across distinct self-concept clarity profiles. The model fit indices for the latent profile analysis of self-concept clarity were shown in Table 3. In the study, latent profile models with two to six profiles were sequentially tested. The results indicated that the entropy for the three-profile model was greater than 0.8 (Clark & Muthén, 2009), and the reductions in AIC, BIC, and aBIC were most pronounced at three profiles, which was the inflection point (Nylund-Gibson et al., 2023). For the three-profile model, the probability of classifying each observation into a latent profile ranged from 0.90 to 0.92, indicating high classification accuracy. Therefore, the three-profile model was determined to be the optimal latent profile model.

**Figure 1:**
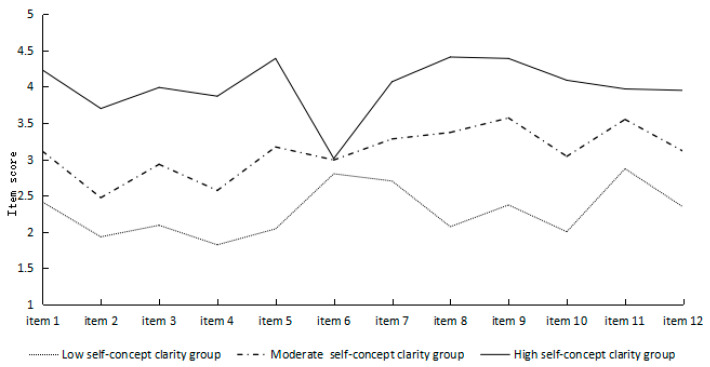
Latent subgroups of adolescent self-concept clarity.

As shown in Figure 1, the first group consisted of 653 adolescents, accounting for 28.54% of the total sample. The group scored lower on the self-concept clarity and was labeled as the low self-concept clarity group. The second group included 1221 adolescents, making up 53.37% of the total sample. The group scored at a moderate level on self-concept clarity and was labeled as the moderate self-concept clarity group. The third group consisted of 414 adolescents, accounting for 18.09% of the total sample. Except for relatively low scores on the sixth item (I rarely feel a conflict between different aspects of my personality), the group scored high on other items, and was labeled as the high self-concept clarity group.

Using the results of potential profile analysis as the dependent variable, with the low self-concept clarity group as the reference profile, and age stage (with junior middle school students as the reference) and gender (with male adolescents as the reference) as independent variables, a logistic regression was conducted to analyze the Odd Ratio (OR) coefficients, reflecting the probabilities of different age stages and genders on their respective self-concept clarity potential profiles. The results were as follows: compared to junior middle school students, there was a higher proportion of senior high school (OR = 1.35, *p* < 0.001, 95%CI = [1.05–1.73]) and college students (OR = 1.82, *p* < 0.001, 95%CI = [1.44–2.41]) in the moderate self-concept clarity group. A similar result was also observed in the high self-concept clarity group where senior high school (OR = 1.20, *p* < 0.001, 95%CI = [0.81–1.77]) and college students (OR = 3.78, *p* < 0.001, 95%CI = [2.72–5.27]) also exhibited a higher proportion. The results indicated that compared to junior middle school students, senior high school and college students were more likely to belong to the moderate or high self-concept clarity group. In addition, compared to male adolescents, the proportion of female adolescents in the moderate self-concept clarity group was lower (OR = 0.75, *p* < 0.05, 95%CI = [0.62–0.92]), and the proportion of female adolescents in the high self-concept clarity group was also lower (OR = 0.47, *p* < 0.001, 95%CI = [0.37–0.61]). The results indicated that, compared to male adolescents, female adolescents were more likely to belong to the lower self-concept clarity group. All results suggest that age and gender are effective predictive variables for the potential profiles of self-concept clarity.

### 3.4. The Relationship Between the Profile of Self-Concept Clarity in Adolescents and the Meaning in Life

As shown in Table 4, the results of the group comparison test indicated significant differences in meaning in life among adolescents with the three profiles of self-concept clarity. Through Bonferroni multiple comparisons, it was found that the high self-concept clarity group had the highest meaning in life (5.90 ± 0.93), coherence (5.83 ± 0.98), purpose (5.92 ± 1.12), and significance (5.93 ± 1.03), followed by the moderate self-concept clarity group, while the low self-concept clarity group had the lowest scores.

## 4. Discussion

### 4.1. The Relationship Between Adolescents’ Self-Concept Clarity and Meaning in Life from Variable-Centered Perspective

The study explored the developmental characteristics and relationships of adolescents’ self-concept clarity and meaning in life from a variable-centered perspective. The results showed that, whether in overall meaning in life or in the dimensions of coherence, purpose, and significance, self-concept clarity was significantly positively correlated with adolescents’ meaning in life. This validated H_1_ and was consistent with previous research findings on the two-dimensional meaning in life (Błażek & Besta, 2012; Church et al., 2014; Han, 2023; Hasson-Ohayon et al., 2014; Liu et al., 2023; Mai et al., 2023). The study is the first to simultaneously validate that adolescents with higher self-concept clarity have higher meaning in life throughout the entire adolescent stage (including early, middle, and late adolescence), highlighting the stability of the relationship between the two. In addition, there were differences in self-concept clarity among adolescents based on age and gender, which was consistent with previous research (Crocetti et al., 2016; Xiang, 2022; Xiang et al., 2023). The self-concept clarity in junior middle school students is lower than that in college students. In other words, over the course of adolescence, the self-concept clarity in adolescents gradually improves, which to some extent verifies that the self-concept clarity is in a continuous upward trend during this stage (Crocetti et al., 2016; Shin et al., 2016; Wu et al., 2010). The gradual exploration of self-identity and the deepening of self-awareness in adolescents are related. As adolescents mature, they become increasingly capable of clearly identifying and integrating their behaviors across different social roles, thereby facilitating the development of a more coherent and stable self-concept. Additionally, male adolescents have a higher self-concept clarity compared to female adolescents. Previous studies have also shown that female adolescents have lower self-esteem, less stable self-concepts, and lower confidence (Xiang et al., 2021). The reason might be related to societal gender expectations. Males are more likely to learn how to expand themselves, take risks, and achieve goals, and these endeavors might contribute to the development of self-concordance (Elliott, 1988). In contrast, female adolescents tend to place greater emphasis on interpersonal relationships than males do (Joshanloo, 2018), and their self-perception is often influenced by others, which might slow down the development of their self-concept.

Adolescents’ meaning in life also varies by age and gender. In terms of age differences, senior high school students reported the lowest overall meaning in life, coherence, and purpose, with junior middle school students scoring higher than senior high school students but lower than college students. Regarding the perceived importance of meaning, there was no significant difference between junior high and senior high school students, but both groups scored significantly lower than college students. In brief, adolescents in early and middle adolescence show a lower meaning in life compared to those in late adolescence. The study partially confirms that the meaning in life tends to increase dynamically with age during adolescence (Y. Li, 2023), which is generally consistent with the views of Erikson (1968) and Battista and Almond (1973). In other words, adolescents in early and middle adolescence often experience a coexistence of developing identity and frequent psychological challenges, which might make them more susceptible to a relatively lower meaning in life (Erikson, 1968). By contrast, college students in late adolescence typically begin to adopt a more active life orientation. They are more likely to construct a meaning in life through commitment to life goals, the pursuit and attainment of personal aspirations, and deep reflection on life’s significance, thereby enhancing their overall sense of meaning (Battista & Almond, 1973). However, unlike previous studies, the present research did not find a marked increase in the meaning in life during middle adolescence (Wang et al., 2016). Interestingly, the results indicated that adolescents in middle adolescence—particularly those in senior high school—demonstrated the lowest levels of meaning in life compared to their early and late adolescent counterparts. This finding may be closely related to the increasingly severe mental health issues faced by senior high school students in China. A meta-analysis has indicated a temporal deterioration in psychological well-being within this population, with anxiety symptoms being especially pronounced (Yu et al., 2022). The relatively weak association between age and meaning in life observed in this stage might reflect the significant challenges senior high school students face in developing meaning in life. With regard to gender differences, male adolescents reported higher overall levels of meaning in life, coherence, and purpose compared to their female counterparts; however, no significant gender difference was found in the perceived importance of meaning. Although the study employed a different measurement tool from previous research to assess the dimensions of meaning in life, the findings are largely consistent with earlier studies (P. Zhang et al., 2022), particularly in the cognitive dimension (coherence) and the motivational dimension (purpose). During adolescence, males tend to perceive greater connectedness among the self, others, events, and various life elements, and they are more capable of integrating past experiences, present circumstances, and future aspirations into a coherent whole, which aligns with their cognitive characteristics. Furthermore, male adolescents scored higher than female adolescents on the dimension of purpose, which might be closely related to differing societal expectations regarding the life goals of male adolescents and female adolescents.

While variable-centered approaches have provided valuable insights into the relationship between self-concept clarity and adolescents’ meaning in life, such approaches often fall short in capturing intra-individual differences and might overlook important information. Therefore, the following analysis adopts the person-centered perspective to identify the demographic characteristics (e.g., age and gender) of adolescents with different profiles of self-concept clarity, and to explore how these profiles relate to their meaning in life.

### 4.2. The Relationship Between Adolescents’ Self-Concept Clarity and Meaning in Life from Person-Centered Perspective

The study adopted a person-centered approach to explore the developmental characteristics and interrelations between self-concept clarity and meaning in life among adolescents. The findings supported H_3_, indicating that distinct profiles of self-concept clarity exist during adolescence, and that individuals with different profiles exhibit significant differences in their meaning in life.

Specifically, three profiles of self-concept clarity were identified: low self-concept clarity group, moderate self-concept clarity group, and high self-concept clarity group. Among these, the high self-concept clarity group accounted for the smallest proportion (18.09%), while the moderate group was the largest (52.96%). Nearly 30% of adolescents (28.54%) were classified in the low self-concept clarity group. This differentiation of “profiles” further validates the person–context interaction theory (Magnusson & Stattin, 1998), suggesting that the development of self-concept clarity exhibits a diverse pattern. During their self-exploration process, adolescents go through different identity statuses. The low self-concept clarity group may be in an “identity confusion” stage, the high self-concept clarity group may be in an “identity establishment” stage, and the moderate self-concept clarity group may be in a “transitional” stage. The results are consistent with prior study (Xiang et al., 2021), showing a U-shaped distribution of adolescent self-concept clarity, with the majority of adolescents falling in the moderate self-concept clarity profile. This indicates that more than half of adolescents are still in the process of developing their self-concept, and they continue to face the task of refining their self-identity.

In addition, the study found that age group and gender significantly predicted membership in the profiles of self-concept clarity, further supporting previous research (Xiang, 2022). Specifically, compared to junior middle school students, senior high school students and college students were more likely to belong to the moderate or high self-concept clarity groups. Male adolescents were also more likely than female adolescents to be classified in the higher self-concept clarity profiles. These findings are consistent with those obtained from variable-centered analyses, reinforcing the notion that both age and gender influence not only the level of self-concept clarity but also its latent profiles (Xiang et al., 2023). In other words, adolescents at different developmental stages and of different genders might exhibit distinct characteristics and tendencies in the construction and maintenance of self-concept.

Finally, differences were found in overall meaning in life and its dimensions across the different profiles of self-concept clarity, with the high self-concept clarity group reporting the highest levels of meaning in life. This suggests that the profiles of self-concept clarity play an important role in shaping adolescents’ meaning in life. When individuals possess a higher level of self-concept clarity, they are more capable of engaging in self-expansion and understanding the connections between their experiences and behaviors (Emery et al., 2015). Self-concept clarity influences adolescents’ outcome expectations, goal selection, and goal pursuit strategies, making them less susceptible to external disturbances (Jiang et al., 2023; Massey et al., 2008). Moreover, it facilitates a deeper understanding of one’s uniqueness, authenticity, and the significance of one’s existence (Ferris et al., 2009; Koriat, 2012; B. Zhang & Lin, 2023). In addition, individuals in the low self-concept clarity profile had the lowest meaning in life, which suggested that teachers and parents should pay special attention to this group of students. Schools and families can serve as key support systems, providing an environment conducive to adolescents’ self-exploration, helping them enhance their self-concept clarity, and thereby gradually improving their meaning in life as they grow.

### 4.3. Implications and Limitations

The study, utilizing cross-sectional data, elucidated the relationship between adolescents’ self-concept clarity and meaning in life through both correlation and latent profile analyses. The correlation analysis confirmed a stable positive association between self-concept clarity and meaning in life throughout adolescence, emphasizing the pivotal role of self-concept clarity as a core component of the individual’s self-structure in the development of life meaning. Latent profile analysis revealed multiple subgroups of adolescents characterized by distinct levels of self-concept clarity, further highlighting the significance of individual differences in shaping adolescents’ experience of meaning in life. The identification of such group heterogeneity facilitates more precise mental health assessments and stratified interventions, providing practical pathways for educational practice. However, the study still has some limitations. First, a cross-sectional study cannot establish causal directions between variables, limiting mechanistic explanations of how self-concept clarity influences meaning in life. Second, the lack of tracking intra-individual dynamic changes prevents examination of fluctuations and mutual influences of self-concept clarity and meaning in life over time. Third, cross-sectional data might be affected by confounding variables, compromising the internal validity of the findings. Future research should adopt more diverse methodologies to thoroughly explore the psychological mechanisms underlying the relationship between these constructs, further enrich theoretical models of self-concept clarity and meaning in life, and guide more effective personalized interventions.

## 5. Conclusions

Several important conclusions emerged from the study. Adolescents’ self-concept clarity is significantly and positively associated with their meaning in life and its various dimensions. Three distinct profiles of self-concept clarity were identified: high, moderate, and low. Both age and gender were significant predictors of adolescents’ membership in these self-concept clarity profiles. Furthermore, the high self-concept clarity group exhibited the highest levels of overall meaning in life, coherence, purpose, and significance, followed by the moderate group, and the low group scoring the lowest across all dimensions.

## Figures and Tables

**Table 1:** Developmental characteristics of adolescents’ self-concept clarity and meaning in Life.

	Age Groups			*F*	Gender		*t*
Variables	Junior Middle School Students	Senior High School Students	College Students		Male	Female	
self-concept clarity	2.89 ± 0.64	2.90 ± 0.57	3.17 ± 0.70	51.51 **	3.12 ± 0.68	2.96 ± 0.65	6.02 **
meaning in life	4.99 ± 1.21	4.76 ± 1.10	5.33 ± 1.10	53.78 **	5.17 ± 1.16	5.03 ± 1.14	2.77 **
coherence	5.06 ± 1.25	4.65 ± 1.10	5.25 ± 1.11	55.66 **	5.16 ± 1.19	4.94 ± 1.14	4.55 **
purpose	5.06 ± 1.35	4.73 ± 1.34	5.41 ± 1.22	57.49 **	5.23 ± 1.33	5.07 ± 1.31	3.02 **
significance	4.87 ± 1.39	4.90 ± 1.33	5.32 ± 1.27	31.73 **	5.11 ± 1.32	5.09 ± 1.34	0.22

Note: ** stands for *p* < 0.01.

**Table 2:** Correlations between self-concept clarity and meaning in life.

Variables	Junior Middle School Students				Senior High School Students				College Students			
	1	2	3	4	1	2	3	4	1	2	3	4
1. self-concept clarity	1 **				1				1			
2. meaning in life	0.49 **	1			0.45 **	1			0.45 **	1		
3. coherence	0.46 **	0.92 **	1		0.42 **	0.90 **	1		0.47 **	0.93 **	1	
4. purpose	0.39 **	0.90 **	0.75 **	1	0.35 **	0.87 **	0.71 **	1	0.38 **	0.92 **	0.80 **	1
5. significance	0.47 **	0.91 **	0.75 **	0.70 **	0.42 **	0.86 **	0.70 **	0.56 **	0.41 **	0.91 **	0.76 **	0.72 **

Note: ** stands for *p* < 0.01.

**Table 3:** Fit indices of latent profile analysis for self-concept clarity.

M	K	AIC	BIC	aBIC	LMR	Entropy	Class Ratio
2	37	80,604	80,816	80,698	4527 ***	0.816	61.50/38.50
3	50	79,173	79,460	79,301	1442 ***	0.810	28.54/53.37/18.09
4	63	78,826	79,187	78,987	369 ***	0.764	46.85/18.27/25.92/8.96
5	76	78,550	78,986	78,745	159 ***	0.774	17.52/45.50/14.34/14.90/7.74
6	89	78,409	78,920	78,637	165 **	0.733	19.80/26.75/14.51/18.96/12.68/7.30

Note: M stands for model, K represents degrees of freedom, AIC stands for Akaike Information Criterion, BIC stands for Bayesian Information Criterion, aBIC stands for sample size adjusted Bayesian Information Criterion, LMR stands for Likelihood Ratio Index, Entropy represents entropy, and Class Ratio represents the proportion of each class of students. ** stands for *p* < 0.01, *** stands for *p* < 0.001.

**Table 4:** Differences in meaning in life across profiles of self-concept clarity.

Variables	Low Self-Concept Clarity Group (*M* ± *SD*)	Moderate Self-Concept Clarity Group (*M* ± *SD*)	High Self-Concept Clarity Group (*M* ± *SD*)	*F*	*p*	$\eta_{p}^{2}$	Multiple Comparison
meaning in life	4.53 ± 1.23	5.12 ± 1.00	5.90 ± 0.93	210.55 **	<0.01	0.16	low < moderate < high
coherence	4.48 ± 1.24	5.06 ± 1.01	5.83 ± 0.98	199.07 **	<0.01	0.15	low < moderate < high
purpose	4.65 ± 1.43	5.13 ± 1.18	5.92 ± 1.12	131.05 **	<0.01	0.10	low < moderate < high
significance	4.46 ± 1.41	5.16 ± 1.20	5.93 ± 1.03	181.20 **	<0.01	0.14	low < moderate < high

Note: ** stands for *p* < 0.01.

## Data Availability

The datasets processed and analyzed during the current study are available from the corresponding/first author upon reasonable request. The data are not publicly available due to their containing information that might compromise the participants’ privacy.
